# Real World Experience of Daratumumab: Evaluating Lymphopenia and Adverse Events in Multiple Myeloma Patients

**DOI:** 10.3389/fonc.2020.575168

**Published:** 2021-02-15

**Authors:** Francesca Cottini, Ying Huang, Nita Williams, Naresh Bumma, Abdullah M. Khan, Maria Chaudhry, Srinivas Devarakonda, Yvonne A. Efebera, Don M. Benson, Ashley E. Rosko

**Affiliations:** Division of Hematology, Department of Internal Medicine, The Ohio State University Comprehensive Cancer Center, Columbus, OH, United States

**Keywords:** myeloma, daratumumab, lymphopenia, infections, outcomes

## Abstract

Multiple myeloma (MM) is an incurable disease with a limited life expectancy of five years from diagnosis. Uncontrolled disease or infections are the main causes of mortality. Daratumumab, a monoclonal antibody against CD38, is approved to treat patients with MM. Its target, CD38, is expressed not only on MM cells but also on common lymphoid precursors and subsets of normal lymphocytes. Daratumumab-induced lymphopenia is common, but its clinical significance is understudied. In this study, we report the baseline characteristics, rates of severe lymphopenia, infections, and clinical trajectory of multiple myeloma patients (n = 100) treated with daratumumab-based regimens at the Ohio State University Comprehensive Cancer Center. We discover high rates of infections, hospital utilization, and severe lymphopenia and identify risks factors for severe lymphopenia, such as low pretreatment absolute lymphocyte count (ALC) values. Severe lymphopenia persists in 23% of patients, resulting in worst survival outcomes. Our data underline the importance of monitoring ALC and consider future use of prophylactic measures or alternative regimens in subsets of MM patients.

## Introduction

Multiple myeloma (MM) is an incurable disease with a limited life expectancy of five years from diagnosis. MM can present acutely with critical illness and multi-organ impairment, leading to morbidity and mortality. Death from MM is secondary to infections or uncontrolled disease (1). The immune system of MM patients is compromised, increasing risk of infections but also hindering anti-tumoral responses (2, 3). Treatment of MM has dramatically evolved in the past ten years. Daratumumab, an IgG kappa monoclonal antibody against CD38, is commonly used in patients with MM (4–8), with overall response rates (ORR) ranging from 40-90% of relapsed/refractory MM patients. Daratumumab targets CD38, an ectoenzyme expressed on the surface of malignant plasma cells but also on common lymphoid precursors and subsets of mature lymphoid cells. Therapy-induced lymphopenia has been reported in 15-25% of patients; however, its clinical significance is vastly unexplored. Here, we report the baseline MM characteristics and clinical course of one hundred (n = 100) MM patients treated with daratumumab-based regimens at the Ohio State University Comprehensive Cancer Center from November 2015 to November 2019. We define the rate of total infections, serious infections, and lymphopenia, identify risks factors for severe lymphopenia, and correlate with patient outcomes.

## Methods

### Patients and Disease Characteristics

The patients described in this article were enrolled in a retrospective single-center study, approved by the Ohio State University Institutional Review Board (2017C0101). Patients were included in the conducted research after written informed consent for the Ohio State University MM registry was obtained (OSU-10115). Patient and disease characteristics of one hundred (n = 100) MM patients who received daratumumab-based regimens at the Ohio State University Comprehensive Cancer Center from November 2015 to November 2019 were retrospectively collected from the medical records. All the patients had a diagnosis of MM, according to International Myeloma Working Group (IMWG) criteria. Patients were included in the analysis if they had received and completed daratumumab-based regimens during the time of evaluation. Per institutional policy, all MM patients had complete blood count, with white blood cell differential, absolute lymphocyte count (ALC), and absolute neutrophil count (ANC) values available, at day of starting daratumumab (pretreatment) and the day of each daratumumab infusion. Stage and cytogenetics were also available at diagnosis.

### Grading of Lymphopenia and Neutropenia and Definition of Infections

Lymphopenia was defined as ALC values (remove absolute lymphocyte counts) below the lower limit of normal (LLN = 1,000 lymphocytes/μl). We followed the Common Terminology Criteria for Adverse Events (CTCAE) Version 5.0 to classify grading of lymphopenia and neutropenia. Specifically, ALC values higher than 1,000 lymphocytes/μl are considered non lymphopenic (Grade 0); ALC values of 1,000–800 lymphocytes/μl are scored as Grade 1 lymphopenia; ALC values of 500–800 lymphocytes/μl are scored as Grade 2 lymphopenia; ALC values of 200–500 lymphocytes/μl are scored as Grade 3 lymphopenia; and ALC values of <200 lymphocytes/μl are scored as Grade 4 lymphopenia. Any ALC value less or equal to 500 lymphocytes/μl is called severe lymphopenia in the manuscript. Similarly, neutropenia is any ANC value less than or equal to 1,500 neutrophils/μl while severe neutropenia any ANC value less than or equal to 500 neutrophils/μl.

Infectious diseases are classified based on the World Health Organization’s International Statistical Classification of Diseases–10th Revision (ICD-10). Infections were divided in non-serious infections or serious infections, with the latter requiring hospitalization.

Serious infections belong to four categories: pneumonia, sepsis, urinary tract infections, and other. Pneumonia are defined by clinical features, radiological findings, as well as positive sputum cultures, polymerase chain reaction (PCR) testing, or serum antigens (9). Patients with pneumonia could have a positive viral culture (e.g. Influenza A/B) but also have a defined consolidation by imaging. Culture positive infections are serious infections with an associated causative agent. This agent is either bacterial, viral, or fungal and is identified by positive microbial culture, PCR, or antigen detection (e.g. *Streptococcus pneumoniae* antigen, Fungitell β-D Glucan assay). Sites of positive cultures are blood (e.g. *Streptococcus pneumoniae* antigen, *Escherichia coli* blood culture), upper or lower respiratory tract secretions, or urine.

Non-serious infections are categorized into upper respiratory tract infections, urinary tract infections, diarrhea disease, and skin infections. Non-infectious related hospital stays are the number of hospital admissions due to other causes than infections.

### Treatment Plan and Response Criteria

Patients received daratumumab at a dose of 16 mg per kilogram intravenously weekly (on days 1, 8, 15, and 22) for 8 weeks, every 2 weeks (on days 1 and 15) for 16 weeks, and every 4 weeks thereafter. Immunomodulatory drugs (IMIDs) were given orally from Day 1–21 on 28-day cycle at 10 to 15 mg daily for lenalidomide or 2 to 4 mg daily for pomalidomide. Bortezomib was given subcutaneously weekly or bi-weekly at 1.3 to 1.0 mg per square meter.

Response criteria and disease progression were defined accordingly to the International Myeloma Working Group (IMWG) criteria. Overall response rates included stringent complete responses (sCR), complete responses (CR), very good partial responses (VGPR), and partial responses (PR). Non responder patients are those with minimal response (MR), stable disease (SD), or progressive disease (PD) to therapy.

### Statistical Analysis

Our primary endpoints were rates of serious and non-serious infections, and severe lymphopenia. Secondary endpoints included overall response rates, progression-free survival (PFS), overall survival (OS), and predictors of severe lymphopenia development. Descriptive statistics, such as medians and ranges for continuous variables and counts and percentages for categorical variables, were used to summarize patient demographics, disease characteristics, and therapy-related serious and non-serious infections. To compare characteristics between patients with severe lymphopenia (ALC ≤ 500) and those without severe lymphopenia (ALC >500) Fisher’s exact test or chi-square χ^2^ test were used for categorical variables, while the Wilcoxon rank-sum test was used for continuous variables. Logistic regression was used to assess the risk of developing pneumonia and extramedullary MM (EMM). PFS and OS were measured from starting date of daratumumab to disease progression or death for PFS, and to death due to all causes for OS; patients without events were censored at time of last follow-up. PFS and OS were estimated using the Kaplan-Meier method, and the log-rank test was used to test the statistical differences between survival curves. To estimate the effect of lymphopenia on survival outcomes, the Cox regression models were fit with backward selection method to build the final multivariable model. In the regression models, the lowest ALC value was included as a continuous variable, and recovery of lymphopenia was included as a time-dependent variable. Two-sided p-values <0.05 were considered statistically significant. Statistical analyses were performed using SAS software version 9.4.

## Results

### Patients Characteristics

From November 2015 to November 2019, 100 MM patients completed daratumumab-based regimens at the Ohio State University Comprehensive Cancer Center. Their clinical and disease characteristics were analyzed retrospectively and are presented in **Table 1**. The majority of patients were male (57%) and White/Caucasian (84%). Nineteen percent of the patients had IgA disease; a total of 69% of patients had stage II or III disease, and 28% had a high-risk cytogenetic profile, defined by the presence of t(4;14), t(14;16), t(14;20), del(17p), or hypodiploid karyotype. The median number of prior regimens was three (range: 0 to 12). The majority of patients (70%) have undergone autologous stem cell transplant (ASCT) prior to daratumumab therapy, with three patients receiving more than one transplant. All but four of the patients have previously received both lenalidomide and bortezomib; 77% of the patients have received additional therapies including pomalidomide, carfilzomib, ixazomib, or other agents prior to daratumumab. At initiation of daratumumab, the median pretreatment ALC value was 1,005 lymphocytes/μl (range: 280 to 3,610), while the median ANC value was 2,410 neutrophils/μl (range: 110 to 15,070). Fifty patients (50%) started daratumumab therapy with at least grade 1 lymphopenia (10 of them with severe lymphopenia) and eighteen patients (18%) with any grade of neutropenia. In patients with more than three prior lines of therapy, pretreatment ALC values were similar (median: 900, range: 300 to 2,970) to patients treated with less than or equal to three lines of therapy (median: 1,140; range: 280 to 3,610) (p-value = 0.35). Twenty-six of our patients received daratumumab as a single agent, fifty-five were treated in combination with immunomodulatory drugs (IMIDs), and sixteen in combination with proteasome inhibitors (PIs). Median pretreatment ALC values were 995, 1,000, and 845 lymphocytes/μl, respectively. All but five patients received anti-viral prophylaxis, with acyclovir or valacyclovir. No other anti-microbial prophylaxis was routinely administred, including no anti-cytomegalovirus (CMV) prophylaxis (letermovir) or anti-bacterial prophylaxis (azithromycin or levofloxacin). Patients received daratumumab-based regimens for a median of 5.1 months (range: 0.5 to 37.2 months). The main reason for treatment discontinuation was disease progression (94/100), with infections, death-related infections, or patient preference accounting for 6% (6/100) of treatment discontinuations.

**Table 1:** Patient demographics, clinical characteristics, and outcomes (n = 100).

	Overall (n = 100)	ALC ≤500 (n = 59)	ALC >500 (n = 41)	p-value
Lowest ALC, Median (range)	405 (0–2,250)	300 (0–500)	750 (550–2,250)	NA
Age, Median (range)	67 (38–90)	66 (38–89)	67 (43–90)	0.65
Gender- no (%) Male Female	57 (57) 43 (43)	39 (66) 20 (34)	18 (44) 23 (56)	0.04
Race or Ethnicity- no (%) White/Caucasian African American	84 (84) 16 (16)	54 (92) 5 (8)	30 (73) 11 (27)	0.02
MM type - no (%) IgG IgA LLC/KLC Other	57 (57) 19 (19) 19 (19) 5 (5)	28 (47) 14 (24) 14 (24) 3 (5)	29 (71) 5 (12) 5 (12) 2 (5)	0.12
ISS – no (%) I II– III NA	30 (31) 66 (69) 4	17 (30) 39 (70) 3	13 (33) 27 (68) 1	0.83
Cytogenetic risk profile – no (%) Standard High NA	66 (72) 26 (28) 8	38 (69) 17 (31) 4	28 (76) 9 (24) 4	0.64
Number of prior treatments Median (range)	3 (0–12)	3 (0–12)	4 (1–10)	0.96
Prior transplant- no (%) Yes No	70 (70) 30 (30)	43 (73) 16 (27)	27 (66) 14 (34)	0.51
Combination – no (%) Single agent Proteasome inhibitors (PIs) Immunomodulatory drugs (IMIDs) Other	26 (26) 16 (16) 55 (55) 3 (3)	11 (19) 9 (15) 37 (63) 2 (3)	15 (37) 7 (17) 18 (44) 1 (2)	0.18
Combination – no (%) Single agent All other combinations	26 (26) 74 (74)	11 (19) 48 (81)	15 (37) 26 (63)	0.04
Pretreatment ALC (at Dara starting date) Median (range)	1,005 (280–3,610)	730 (280–2,440)	1460 (650–3,610)	<0.0001
Pretreatment ANC (at Dara starting date), Median (range)	2,410 (110–15,070)	2,110 (110–15,070)	2,700 (460–9,200)	0.15
Lowest ANC, Median (range)	1,115 (100–5,700)	960 (110–3,100)	1,940 (100–5,700)	0.002
Cumulative dexamethasone dose (mg), Median (range)	202 (12–880)	180 (12–880)	228 (20–640)	0.85
ORR	54 (54)	30 (51)	24 (59)	0.54
Total Infections- no (%)*	49 (49)	31 (53)	18 (44)	0.42
Non-infectious related hospital stays- no (%)	32 (32)	18 (31)	14 (34)	0.70
Number of ED Visits, Median (range)	0 (0–7)	0 (0–7)	0 (0–5)	0.22

*Odds ratio for total infections: 0.93 (95% CI: 0.84–1.03, p-value = 0.16). For each 100 lymphocytes/μl increase in the lowest ALC, the odds of all infections decreased by 7%.

ISS, International Staging System. ISS consists of three stages, with higher stages associated with more severe disease: stage I, serum β2-microglobulin level less than 3.5 mg/L and albumin level above 3.5 g/dL; stage II, neither stage I nor III; and stage III, serum β2-microglobulin level more than 5.5 mg/L.

Cytogenetics risk profile. A high-risk cytogenetic profile was defined by a finding of t(4;14), t(14;16), t(14;20), del(17p), or hypodiploid karyotype. All other patients belong to the standard risk group.

ORR, Overall Response Rates include Complete Responses (CR), Very Good Partial Responses (VGPR), and Partial Responses (PR). ED visits, Emergency Department visits. All other combinations include daratumumab in combination with IMIDs, PIs, or other.

### Infections, Rates and Grading of Lymphopenia and Neutropenia in Patients Treated With Daratumumab-Based Regimens

We initially evaluated total infections, serious infections requiring hospitalization, non-serious infections, and grades of lymphopenia and neutropenia in the cohort of 100 MM patients. While on therapy, the total infection rate was 49%. Serious infections occurred in 35 patients, with 12/100 patients requiring multiple hospital admissions for infections. Pneumonia occurred in 23 (23%) patients, followed by sepsis (8 patients—8%), one case of urinary tract infection (1%), and 3 cases (3%) of other infections, such as Epstein-Barr virus (EBV)/CMV reactivation, fungal meningitis, and fevers of unknown origin (**Table 2**). All patients with serious infections presented with clinical, laboratory, and radiological signs of infections, with 24/35 patients (69%) resulting in positive cultures (**Table 2**). Non-serious infections occurred in 14% of the patients, with upper respiratory tract infections accounting for half of the infections, followed by urinary tract infections, diarrhea disease, or skin infections (**Supplementary Table 1**).

**Table 2:** Serious infection characteristics in all patients (n = 100).

	Overall(n = 100)	ALC ≤500(n = 59)	ALC >500(n = 41)	p-value
Serious infections- (%)*	35 (35)	26 (44)	9 (22)	0.02
Pneumonia**	23 (23)	19 (32)	4 (10)	0.01
Sepsis**	8 (8)	4 (7)	4 (10)	0.71
Urinary tract infection	1 (1)	1 (2)	0 (0)	1.00
Other	3 (3)	2 (3)	1 (2)	1.00
	n = 35	n = 26	n = 9	
Culture positive infections- no %	24 (69)	18 (69)	6 (67)	1.00
Bacterial**	13 (37)	9 (35)	4 (44)	0.70
Viral**	10 (29)	8 (31)	2 (22)	1.00
Fungal	1 (3)	1 (4)	0 (0)	1.00
Time to infection in days- Median (range)	56 (6–755)	52 (6–755)	91 (8–298)	0.61
ANC values at time of infection Median (range)	3,580 (550–15,250)	3,950 (700–15,250)	3,290 (550–10,100)	0.52

*Odds ratio for serious infections: 0.84 (95% CI: 0.73–0.96, p-value = 0.01). For each 100 lymphocytes/μl increase in the lowest ALC, the odds of serious infections decreased by 16%.

**Odds ratio for pneumonia: 0.78 (95% CI: 0.64–0.94, p-value = 0.009). For each 100 lymphocytes/μl increase in the lowest ALC, the odds of pneumonia decreased by 22%.

Thirty-two patients were admitted to the hospital for non-infectious related causes. Main reasons for admissions included pain control or fractures, cardiac events, disease progression, infusion reactions, or deconditioning (**Supplementary Table 2**).

The median time from initiation of daratumumab to development of serious infections was 56 days (range: 6 to 755 days), while for non-serious infections was 105 days (range: 3 to 282 days).

**Figure 1:**
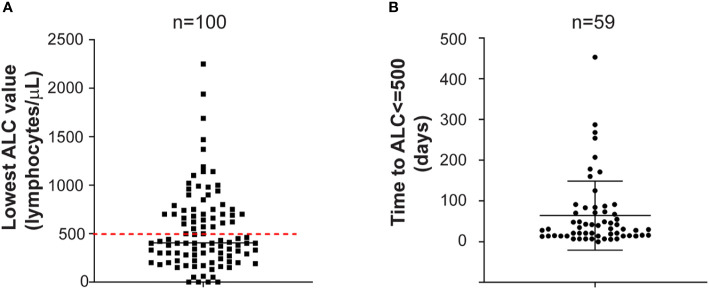
**(A)** Lowest absolute lymphocyte count. Lowest absolute lymphocyte (ALC) value in the entire cohort of MM patients treated with daratumumab-based regimens (n = 100) is shown. Red dashed line is set at 500 lymphocytes/μl, representing the threshold for severe lymphopenia. Mean with standard deviation are shown. **(B)** Time to severe lymphopenia. Time is calculated from ALC at starting daratumumab to ALC less or equal to 500 lymphocytes/μl in the group of MM patients who developed severe lymphopenia (n = 59). Time is in days. Mean with standard deviation are shown.

Among the 100 patients included in this analysis, the lowest median ALC value was 405 lymphocytes/μl (range: 0 to 2250). Ninety percent of the patients developed lymphopenia of any degree. Fifty-nine (59%) developed severe lymphopenia (grade 3–4, median lowest ALC 300 lymphocytes/μl; range: 0 to 500) (**Figure 1A**). Grade 1 lymphopenia occurred in 8 (8%) patients, while grade 2 lymphopenia developed in 23 (23%) patients. The lowest median ANC value was 1,115 neutrophils/μl (range: 100 to 5,700). Neutropenia occurred in 61 (61%) patients, with severe neutropenia happening in 23 (23%) of them. Median ANC value at time of serious infections was 3,580 neutrophils/μl (range: 550 to 15,250). The median lowest ANC value was 950 neutrophils/μl (range: 110 to 4320) in patients treated with daratumumab in combination with IMIDs while was 1900 neutrophils/μl (range: 100 to 5700) in patients treated with daratumumab in combination with PIs or as single agent (p-value = 0.003). Indeed, it is well known that IMIDs can cause neutropenia (10).

Patients with severe lymphopenia had similar rates of *total* infections (53%) compared to patients without severe lymphopenia (44%) (p-value = 0.42). However, patients with severe lymphopenia had higher rates of *serious* infections (44%) compared to 22% of non-severely lymphopenic patients (p-value = 0.02). Severely lymphopenic patients experienced more frequently pneumonia (odds ratio (OR) for pneumonia = 0.78; 95% CI: 0.64–0.94; p-value = 0.009), while similar rates of other types of serious infections occurred (**Table 2**). EBV/CMV reactivation and fungal meningitis happened only in the group of patients with severe lymphopenia. Two infection-related deaths occurred in patients with severe lymphopenia. The median time from initiation of daratumumab to development of serious infections was 52 days (range: 6 to 755 days) for patients with severe lymphopenia and 91 days (range: 8 to 298 days) for patients without severe lymphopenia (p-value = 0.61). Among the 26 severely lymphopenic patients who developed serious infections, 12 patients had infections before the lowest ALC value was measured, despite being already lymphocytopenic, while 14 patients had infections afterwards (median 54, range: 0 to 734). Severely lymphopenic patients had lower median ANC values (960 neutrophils/μl; range: 110 to 3,100) compared to non severely lymphopenic patients (1,940 neutrophils/μl; range: 100 to 5,700) (p-value = 0.002) but similar median ANC values at the time of serious infections (3,950 neutrophils/μl versus 3,290 neutrophils/μl; p-value = 0.52). This suggests that ALC values are the main determinant of infection risk in a subset of patients treated with daratumumab-based regimens.

Interestingly, non severely lymphopenic patients had a trend towards more non-serious infections compared to severely lymphopenic patients (22 vs. 9%, p-value = 0.08) (**Supplementary Table 1**). The median time from initiation of daratumumab to development of non serious infections was 158 days (range: 3 to 282 days) for patients with severe lymphopenia and 99 days (range: 13 to 188 days) for patients without severe lymphopenia (p-value = 0.79).

Non-infectious related hospital stays occurred at similar rates (31 vs. 34% in patients with or without severe lymphopenia; p-value = 0.70).

### Lymphopenia, Neutropenia, and Infections in Patients Treated With Single Agent Daratumumab

We then compared rates of lymphopenia, neutropenia, and infections in patients treated with single agent daratumumab (n = 26) or patients treated with daratumumab in combination with other agents (n = 74). Indeed, also PIs and IMIDs can affect ALC values (11, 12). Single agent daratumumab causes severe lymphopenia less frequently (11/26 patients—42%) than daratumumab in combination with other therapies (48/74—65%). Indeed, among the 59 severely lymphopenic patients, 48 patients (81%) were treated with combination therapy, while among the 41 non severely lymphopenic patients, only 23 patients (63%) were treated with combination therapy (p-value = 0.04). Median lowest ALC values were 640 lymphocytes/μl in patients treated with daratumumab as a single agent and 400 lymphocytes/μl in patients treated with combination therapy (p-value = 0.13), as shown in **Table 3**. The rate of severe neutropenia was lower in patients treated with single agent daratumumab compared to patients treated with combination therapy (15 vs. 26%), although it did not reach statistical significance (p = 0.41). However, looking at the lowest ANC value as a continuous variable, patients treated with single agent daratumumab had significant higher ANC values (1,985 neutrophils/μl) than patients treated with daratumumab in combination with other therapies (970 neutrophils/μl) with p-value of 0.0007.

**Table 3:** Lymphopenia, neutropenia, and infectious characteristics in patients treated with single agent daratumumab (n = 26) or combination (n = 74).

	Single agent (n = 26)				Other combinations (n = 74)				p-value
Lowest ALC Median (range)	640 (150–2,250)				400 (0–1,940)				0.13
Severe Neutropenia, no (%)	4 (15)				19 (26)				0.28
Lowest ANC Median (range)	1,985 (350–5,700)				970 (100–15,070)				0.0007
	**All** **(n = 26)**	**ALC ≤500** **(n = 11)**	**ALC >500** **(n = 15)**	**p-value**	**All** **(n = 74)**	**ALC ≤500** **(n = 48)**	**ALC >500** **(n = 26)**	**p-value**	**p-value**
Serious infections- no (%) Time to infection in days Median (range)	8 (31) 65 (6–238)	6 (55) 51 (6–100)	2 (13) 203 (168–238)	0.04 0.07	27(36) 48 (7–755)	20(42) 66 (7–755)	7 (27) 34 (8–298)	0.21 0.80	0.60 0.88
Non serious infections- no (%) Time to infection in days Median (range)	2 (8) 111 (53–168)	0 (0) NA	2 (13) 111 (53–168)	0.49 NA	12 (16) 105 (3–282)	5 (10) 158 (3–282)	7 (27) 99 (13–188)	0.10 0.75	0.35 0.78

We then evaluated the rates of serious and non-serious infections in patients treated with single agent daratumumab or combination therapy. There was no statistically significant difference in terms of rates of serious infections (p-value = 0.60) or non-serious (p-value = 0.35) infections between patients in the two cohorts (**Table 3**). The median time from initiation of daratumumab to development of serious infections was 65 days (range: 6 to 238 days) for patients treated with single agent daratumumab and 48 days (range: 7 to 755 days) for patients treated with combination therapy (p-value = 0.88). Similarly, there was no statistically significant difference (p-value = 0.78) in the timing of non-serious infections in the two cohorts (111 days versus 105 days).

In patients treated with daratumumab single agent, the severely lymphopenic patients had a much higher rate of serious infections (55%) compared to those who did not develop severe lymphopenia (13%) (p-value = 0.04). Conversely, this comparison was not significant in patients treated with combination therapy (42 vs. 27%, p-value = 0.21). This data confirms that single agent daratumumab can cause lymphopenia and serious infections in a subset of MM patients.

### Timing to Severe Lymphopenia

The median time to severe lymphopenia was 31 days (range: 0 to 453) (**Figure 1B**). Patients with high stage disease (International Staging System-ISS II-III) at diagnosis developed severe lymphopenia quicker than patients with ISS I disease (55 days versus 21 days; p-value = 0.0006). Moreover, patients treated with daratumumab as single agent or in combination with IMIDs developed severe lymphopenia sooner compared to patients treated with daratumumab in combination with PIs (21 days versus 28 days versus 84 days; p-value = 0.06).

### Factors Associated to the Development of Severe Lymphopenia

Among baseline patient and disease characteristics, male gender (p-value = 0.04), Caucasian ethnicity (p-value = 0.02), and pretreatment ALC value at starting of daratumumab (p-value < 0.0001) were associated with risk of severe lymphopenia. Conversely, MM type and stage, age, cytogenetic risk profile, number of prior lines of treatment, prior ASCT, and cumulative dexamethasone dose were not statistically associated with risk of lymphopenia development. Lymphopenia development occurred independently of responses to therapy. Indeed, there was no statistically significant difference in terms of ORR at times of nadir of lymphopenia among the two groups (p-value = 1.0).

### Persistence of Severe Lymphopenia in Daratumumab-Treated Patients

**Figure 2:**
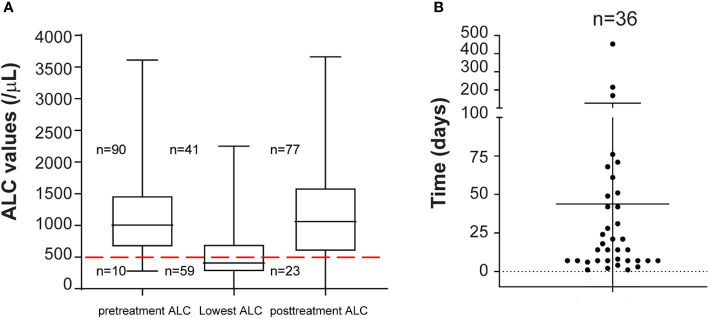
**(A)** ALC recovery kinetics in patients with severe lymphopenia. Pretreatment ALC, lowest ALC and ALC at completion of daratumumab therapy (posttreatment ALC) for all patients (n = 100) are shown. Median and range are plotted. Red dashed line is set at 500 lymphocytes/μl. 10, 59, and 23 patients out of 100 had severe lymphopenia pretreatment, at nadir, and at completion of daratumumab therapy. **(B)** Time to ALC recovery. Patients with ALC recovery above 500 lymphocytes/μl (n = 36) from lowest ALC are shown. Time is in days.

We then followed ALC values of the 59 MM patients treated with daratumumab-based regimens who developed severe lymphopenia. Generally, ALC values were obtained weekly during the first two months of therapy, then twice a month, and then monthly. Some patients had additional ALC values available. We observed that severe lymphopenia is a dynamic process. Sixty-one percent of severely lymphopenic patients (36/59) recovered to ALC values more than 500 lymphocytes/μl, with sixteen of them recovering to ALC values more than 1,000 lymphocytes/μl (**Figure 2A**), while still receiving daratumumab. The median time to ALC recovery above 500 lymphocytes/μl was 14 days (range: 1 to 453) (**Figure 2B**). In the univariable analysis including only patients who developed severe lymphopenia, no factors associated with ALC recovery were identified (**Supplementary Table 3**). However, patients treated with daratumumab in combination with PIs had longer recovery time (p = 0.007) than patients treated with single agent or daratumumab in combination with IMIDs (71 days versus 7 days versus 14 days).

### Outcomes

**Figure 3:**
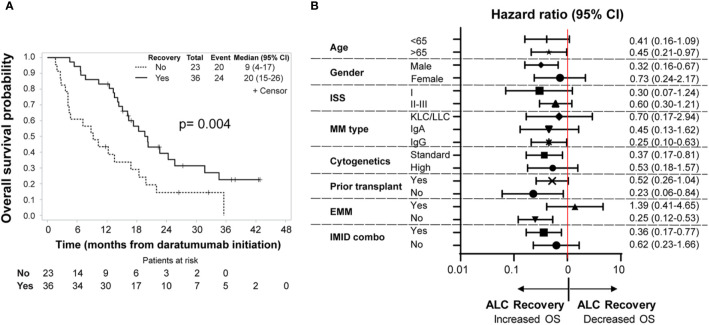
**(A)** Overall survival. Kaplan–Meier analysis of overall survival among patients who developed severe lymphopenia (n = 59). The P value is based on a log−rank test (p-value = 0.004). The solid line represents the ALC recovery group (ALC above or equal to 500 lymphocytes/μl), which includes 36 patients, while the dotted line show the no ALC recovery group, which includes 23 patients. Time is counted in months from day of initiation of daratumumab. **(B)** Subgroup analysis of overall survival in patients with severe lymphopenia. Shown are the hazard ratios of ALC recovery for overall survival in prespecified subgroups of patients with severe lymphopenia (ALC below 500 lymphocytes/μl). n = 59 patients. Hazard ratios and 95% CIs are provided. Time is counted in months from day of initiation of daratumumab. ISS, International Staging System. ISS consists of three stages, with higher stages associated with more severe disease: stage I, serum β2-microglobulin level less than 3.5 mg/L and albumin level more than 3.5 g/dL; stage II, neither stage I nor III; and stage III, serum β2-microglobulin level more than 5.5 mg/L. Cytogenetics risk profile. A high-risk cytogenetic profile was defined by a finding of t(4;14), t(14;16), t(14;20), del(17p), or hypodiploid karyotype. All other patients belong to the standard risk group. EMM, Extramedullary Multiple Myeloma. IMID combo, Immunomodulatory drug- daratumumab combination.

We then evaluated the relationship between severe lymphopenia development and outcomes. We first analyzed outcomes of the entire cohort of 100 MM patients who received daratumumab-based regimens. At a median follow-up of 20.1 (range: 9.2 to 42.8) months, 38 patients were alive. In this analysis of all MM patients, no statistically significant difference in terms of overall response rate (ORR) (51 vs. 59%; p-value = 0.54) (**Table 1**), PFS (p-value = 0.83), or OS (p-value = 0.10) was noted between patients with or without severe lymphopenia (**Supplementary Table 4**). Among the 59 MM patients who developed severe lymphopenia, patients with persistent severe lymphopenia had worse PFS (log rank p = 0.02; median PFS 5.82 months versus 2.76 months) and OS (log rank p = 0.004) compared to patients who recovered their ALC values (**Figure 3A**). The median follow-up for this group was 26.5 months (range: 11.8 to 42.8) among 15 survivors. The effect of recovery was no longer significant for PFS in the multivariable analysis after accounting for other clinical factors. However, it remained a significant prognostic factor for OS (Hazard ratio = 0.42, 95% CI: 0.22-0.78, p-value = 0.006) after controlling for disease type (**Supplementary Table 5**). Furthermore, in the subgroup analysis, patients with male gender, standard cytogenetics, IgG type, age more than 65, and IMID combination who had ALC recovery showed lower risk of death compared to those with persistent lymphopenia (**Figure 3B**). Additionally, patients with severe lymphopenia had higher risk of EMM at relapse after daratumumab (OR of 100 lymphocytes/μl increase in the lowest ALC = 0.82, 95% CI: 0.68–0.99; p-value = 0.03), and the association remained significant even after adjusting for cytogenetic risk profile (OR of 100 lymphocytes/μl increase in the lowest ALC = 0.83, 95% CI: 0.68–1.00, p-value = 0.04).

## Discussion

In summary, this study presents real-world data on rates of infections and severe lymphopenia in patients treated with daratumumab. This is significant and relevant for patient care, since daratumumab is broadly used in relapsed/refractory MM patients but also is tested in clinical trials for other conditions.

A recent metanalysis from Al Hadidi and colleagues (13) evaluated the toxicities of daratumumab as reported in five phase III clinical trials, including the CASTOR (8) and POLLUX trials (4) in relapsed/refractory MM patients, and the ALCYONE, MAIA, and CASSIOPEIA trials (5–7), in newly diagnosed MM patients. In this metanalysis, grade 3–4 neutropenia, grade 3–4 lymphopenia, and pneumonia occurred in 45.9, 13, and 10.6% of patients, respectively. Complementing another recently published retrospective study by Johnsrud and colleagues (14), we report similar rates of neutropenia but higher rates of grade 3–4 lymphopenia (59%), serious infections (35%), and pneumonia (23%), which are even greater in patients with severe lymphopenia. These infections also resulted in two infection-related deaths.

Clinical trials with daratumumab-based regimens have a rate of severe lymphopenia between 5–24%, while severe lymphopenia occurred in 59% of our patients. Several explanations account for this discrepancy. Severe lymphopenia is a dynamic process, happened early on therapy (median time of onset = 31 days), and resolved within two months in 30% of the patients. We were able to detect these changes because we accessed weekly ALC values, while these data might not have been available for clinical trial patients. Our patient population is heavily pre-treated with a median of 3 prior lines of therapies (range: 0 to 12). Moreover, 50% of our patients had at least grade 1 lymphopenia at starting of daratumumab, with pretreatment lymphopenia representing a risk factor for severe lymphopenia.

Lymphopenia is a well-recognized risk factor for infections in MM (15). Grade 3 or 4 lymphopenia occurs with IMIDs and bortezomib (12, 16), at rates of 3 to 6%, as reported in (17, 18). The combination of daratumumab with IMIDs causes higher rates of severe lymphopenia compared to single agent daratumumab or combination with PIs. However, patients treated with single daratumumab who became severely lymphopenic still had higher rates of serious infections compared to patients who did not become severely lymphopenic. In our cohort of patients the median ANC values at time of serious infections were similar among the severely and non-severely lymphopenic patients, indicating that ANC values are not the only determinant of infection development. Moreover, lower ANC values occurred as expected in patients treated with daratumumab in combination with IMIDs (p-value = 0.003).

Currently, anti-viral prophylaxis is recommended with daratumumab therapy independent of ALC values to prevent varicella-zoster virus infection (19). Based on our study, we recommend close monitoring of ALCs, especially for patients with pretreatment non-severe lymphopenia. Since severe lymphopenia is a dynamic process, some patients may recover without additional interventions. Among patients with persistent severe lymphopenia evaluation of CD4/CD8 ratio by flow cytometry and/or antimicrobial prophylaxis with trimethoprim/sulfamethoxazole and azithromycin could be considered with persistently low CD4 count. We also recommend appropriate vaccinations (pneumococcal vaccines and annual influenza vaccines). A recent single institution experience suggested that increased incidence of hypogammaglobulinemia in patients treated with daratumumab (30.3% vs. 61.4%) may lead to increased risk of infections (20). Therefore, consideration of intravenous immunoglobulin (IVIG) replacement for patients with concomitant hypogammaglobulinemia, recurrent life-threatening infections, or in the context of clinical trials should be explored.

Previous studies in MM have showed that ALC values at diagnosis (21, 22) or after ASCT (23–25) are associated with prognosis. Similar findings have been reported in other diseases, such as acute myeloid leukemia, where ALC recovery after induction chemotherapy predicts superior survival (26). Daratumumab induces changes in CD8/CD4 ratios, with reduction of B and T CD38^+^ regulatory cells, naïve T cells (27), and CD38^+^ Natural Killer (NK) cells (28). Patients who respond to daratumumab also have more CD8^+^granzyme B^+^ cytotoxic T cells (29). We identified a trend for shorter PFS and OS in patients with persistent lymphopenia which was no longer significant in the multivariable analysis for PFS, but retained significance for OS. This effect was especially important for subsets of patients, such as patients older than 65, or those receiving dara-IMID combination who might rely even more on intact lymphocyte function to fight infections or promote anti-tumoral immunity. This persistent lymphopenia can indeed increase toxicity or reduce efficacy of sequential lines of therapy. The studies on the prognostic role of lymphopenia are limited to patients at diagnosis or after transplant. Therefore, the relationship between secondary persistent lymphopenia, survival, and disease aggressiveness needs to be addressed in future prospective or retrospective studies.

We have previously reported the impact of transplant with differential effects among T cell subtypes with an exhausted immunophenotype of CD3^+^CD4^+^ subsets and a senescent immunophenotype in CD3^+^CD8^+^ subsets. Daratumumab may further exacerbate T-cell subset defects and result in impaired immunity (30) in certain patients. Changes in these immune populations can account for the development of upper respiratory tract infections, also in patients that are not lymphopenic, as reported in clinical trials (4–8) and in our cohort.

To conclude, the development and persistence of severe lymphopenia in patients receiving daratumumab-based regimens is not uncommon and is associated with risk of serious infections, and worst outcomes in certain subsets of patients. Therefore, close monitoring, prophylactic measures, and consideration of alternative regimens is important, especially in patients with pretreatment lymphopenia.

## Data Availability Statement

The raw data supporting the conclusions of this article will be made available by the authors, without undue reservation.

## Ethics Statement

The studies involving human participants were reviewed and approved by the institutional review board at The Ohio State University (CSRC@osumc.edu): Predictors of response and mechanisms of relapse/progression in daratumumab-treated myeloma (2017C0101); Initial approval: 09/07/2017; Expiration Date: 7/9/2021. Ohio State University Multiple Myeloma and Amyloidosis Data Registry and Sample Resource (B-SCR-MM) (OSU-10115) Initial approval: 3/17/2011; Expiration Date: 10/16/2021. The patients/participants provided their written informed consent to participate in this study.

## Author Contributions

FC performed research, data analysis, and wrote the manuscript, YH performed statistical analysis, NW, NB, AK, MC, SD, YE, and DB consented patients and provided critical review of manuscript. FC, DB, and AR wrote the manuscript. AR supervised the study. All authors contributed to the article and approved the submitted version.

## Funding

This work was supported by NIH grant, K23 CA208010-01 (AR).

## Conflict of Interest

AK is a consultant for Janssen and Amgen. MC is a consultant for Sanofi. NB is on the speaker bureau for Amgen. YE is on the speaker bureau for Takeda and Akcea.

The remaining authors declare that the research was conducted in the absence of any commercial or financial relationships that could be construed as a potential conflict of interest.
