# Supplementary material for: Real World Experience of Daratumumab: Evaluating Lymphopenia and Adverse Events in Multiple Myeloma Patients
Source: Front Oncol. 2021 Feb 15;10:575168. doi: 10.3389/fonc.2020.575168 (PMC7917249; doi:10.3389/fonc.2020.575168)
Supplement: Supplementary file 1 [file DataSheet_1.docx]

**Supplementary Table 1. Non-serious infection characteristics in all patients (n=100)**

|  | Overall  (n=100) | ALC ≤500  (n=59) | ALC >500  (n= 41) | p-value |
| --- | --- | --- | --- | --- |
| All type of non-serious infections- no (%)  Upper respiratory tract infections  Urinary tract infections  Diarrhea disease  Skin infections | 14 (14)  7 (7)  4 (4)  2 (2)  1 (1) | 5 (9)  3 (5)  1 (2)  1 (2)  0 (0) | 9 (22)  4 (10)  3 (7)  1 (2)  1 (2) | 0.08  0.44  0.30  1.00  0.41 |
| Time to infection in days-  Median (range) | 105 (3-282) | 158 (3-282) | 99 (13-188) | 0.79 |

**Supplementary Table 2. Non-infectious related hospital stays in all patients (n=100)**

|  | Overall  (n=100) | ALC ≤500  (n=59) | ALC >500  (n= 41) | p-value |
| --- | --- | --- | --- | --- |
| Non-infectious related hospital stays  Pain control  Cardiac events  Disease progression  Infusion reactions  Deconditioning | 32 (32)  15  5  2  3  7 | 18 (31)  9  1  2  2  4 | 14 (34)  6  4  0  1  3 | 0.70 |

**Supplementary Table 3. Patient characteristics and outcomes by ALC recovery from lymphopenia**

|  | ALC ≤500  (n=59) | Recovery  (n=36) | No recovery  (n= 23) | p-value |
| --- | --- | --- | --- | --- |
| Age, Median (range) | 66 (38-89) | 67 (45-89) | 65 (38-86) | 0.11 |
| Gender- no (%)  Male  Female | 39 (66)  20 (34) | 23 (64)  13 (36) | 16 (70)  7 (30) | 0.78 |
| Race or Ethnicity - no (%)  White/Caucasian  African American | 54 (92)  5 (8) | 33 (92)  3 (8) | 21 (91)  2 (9) | 1.00 |
| MM type - no (%)  IgG  IgA  LLC/KLC  Other | 28 (47)  14 (24)  14 (24)  3 (5) | 18 (50)  7 (19)  10 (28)  1 (3) | 10 (43)  7 (30)  4 (17)  2 (9) | 0.52 |
| ISS – no (%)  I  II- III  NA | 17 (30)  39 (70)  3 | 13 (37)  22 (63)  1 | 4 (19)  17 (81)  2 | 0.23 |
| Cytogenetic Risk profile – no (%)  Standard  High  NA | 38 (69)  17 (31)  4 | 25 (76)  8 (24)  3 | 13 (59)  9 (41)  1 | 0.24 |
| Number of prior treatments  Median (range) | 3 (0-12) | 3 (0-12) | 4 (1-11) | 0.50 |
| Prior transplant- no (%)  Yes  No | 43 (73)  16 (27) | 26 (72)  10 (28) | 17 (74)  6 (26) | 1.00 |
| Combination – no (%)  Single agent  Proteasome inhibitors (PIs)  Immunomodulatory drugs (IMIDs)  Other | 11 (19)  9 (15)  37 (63)  2 (3) | 7 (19)  5 (14)  23 (64)  1 (3) | 4 (17)  4 (17)  14 (61)  1 (4) | 0.96 |
| Baseline ALC (at Dara starting date)  Median (range) | 730 (280-2440) | 870 (300-1700) | 700 (280-2440) | 0.19 |
| Lowest ALC, Median (range) | 300 (0-500) | 330 (0-500) | 220 (0-460) | 0.02 |
| ORR | 30 (51) | 20 (56) | 10 (43) | 0.43 |
| Total Infections- no (%) | 31 (53) | 18 (50) | 13 (57) | 0.79 |
| Serious infections- no (%) | 26 (44) | 16 (44) | 10 (43) | 0.94 |
| Number of ED Visits, Median (range) | 0 (0-7) | 0 (0-7) | 1 (0-3) | 0.28 |

ISS, International Staging System. ISS consists of three stages, with higher stages associated with more severe disease: stage I, serum β2-microglobulin level less than 3.5 mg/L and albumin level above 3.5 g/dL; stage II, neither stage I nor III; and stage III, serum β2-microglobulin level more than 5.5 mg/L.

Cytogenetics risk profile. A high-risk cytogenetic profile was defined by a finding of t(4;14), t(14;16), t(14;20), del(17p), or hypodiploid karyotype. All other patients belongs to the standard risk group.

ORR, Overall Response Rates include Complete Responses (CR), Very Good Partial Responses (VGPR), and Partial Responses (PR). ED visits, Emergency Department visits.

**Supplementary Table 4. PFS and OS by Lymphopenia**

|  | Overall (n=100) | ALC ≤500 (n=59) | ALC >500 (n= 41) | p-value |
| --- | --- | --- | --- | --- |
| PFS  # of events  Median (95% CI) | 100  5.13 (3.68-6.47) | 59  4.47 (2.76-6.47) | 41  6.05 (3.68-7.13) | 0.83 |
| OS  # of events  Median (95% CI) | 62  18.40 (14.13-22.60) | 44  16.36 (13.31-20.17) | 18  26.91 (11.40-NA) | 0.10 |

*Median follow-up: 20.1 months (range: 9.2-42.8) among 38 survivors

**Supplementary Table 5. Multivariable Cox regression model of overall survival among patients who developed severe lymphopenia (n=59).**

| Variable | Hazard Ratio (95% CI) | p-value |
| --- | --- | --- |
| MM Type (vs. IgG)  IgA  KLC, LLC  Other | 0.95 (0.45-2.02)  0.44 (0.19-1.02)  3.01 (0.82-11.04) | 0.05 |
| Recovery from Lymphopenia | 0.42 (0.22-0.78) | 0.006 |

After controlling for the type of MM, patients who recovered from lymphopenia had 58% lower risk for all-cause death compared with patients who never recovered (HR=0.42, p=0.006).
